# Revealing the source of Jupiter’s x-ray auroral flares

**DOI:** 10.1126/sciadv.abf0851

**Published:** 2021-07-09

**Authors:** Zhonghua Yao, William R. Dunn, Emma E. Woodfield, George Clark, Barry H. Mauk, Robert W. Ebert, Denis Grodent, Bertrand Bonfond, Dongxiao Pan, I. Jonathan Rae, Binbin Ni, Ruilong Guo, Graziella Branduardi-Raymont, Affelia D. Wibisono, Pedro Rodriguez, Stavros Kotsiaros, Jan-Uwe Ness, Frederic Allegrini, William S. Kurth, G. Randall Gladstone, Ralph Kraft, Ali H. Sulaiman, Harry Manners, Ravindra T. Desai, Scott J. Bolton

**Affiliations:** 1 Key Laboratory of Earth and Planetary Physics, Institute of Geology and Geophysics, Chinese Academy of Sciences, Beijing, China.; 2 College of Earth and Planetary Sciences, University of Chinese Academy of Sciences, Beijing, China.; 3 Mullard Space Science Laboratory, University College London, Dorking, UK.; 4 Harvard-Smithsonian Center for Astrophysics, Smithsonian Astrophysical Observatory, Cambridge, MA, USA.; 5 The Centre for Planetary Science at UCL/Birkbeck, Gower Street, London WC1E 6BT, UK.; 6 British Antarctic Survey, Cambridge, UK.; 7 Applied Physics Laboratory, Johns Hopkins University, Laurel, MD, USA.; 8 Space Science and Engineering Division, Southwest Research Institute, San Antonio, TX, USA.; 9 Department of Physics and Astronomy, University of Texas at San Antonio, San Antonio, TX, USA.; 10 Laboratoire de Physique Atmosphérique et Planétaire, STAR institute, Université de Liège, Liège, Belgium.; 11 Northumbria University, Newcastle upon Tyne, UK.; 12 Department of Space Physics, School of Electronic Information, Wuhan University, Wuhan, Hubei, China.; 13 CAS Center for Excellence in Comparative Planetology, Hefei, Anhui, China.; 14 European Space Agency (ESA), European Space Astronomy Centre (ESAC), Camino Bajo del Castillo s/n, 28692 Villanueva de la Cañada, Madrid, Spain.; 15 NASA Goddard Space Flight Center, Greenbelt, MD, USA.; 16 Department of Physics and Astronomy, University of Iowa, Iowa City, IA, USA.; 17 Blackett Laboratory, Imperial College London, London, UK.

## Abstract

Jupiter’s rapidly rotating, strong magnetic field provides a natural laboratory that is key to understanding the dynamics of high-energy plasmas. Spectacular auroral x-ray flares are diagnostic of the most energetic processes governing magnetospheres but seemingly unique to Jupiter. Since their discovery 40 years ago, the processes that produce Jupiter’s x-ray flares have remained unknown. Here, we report simultaneous in situ satellite and space-based telescope observations that reveal the processes that produce Jupiter’s x-ray flares, showing surprising similarities to terrestrial ion aurora. Planetary-scale electromagnetic waves are observed to modulate electromagnetic ion cyclotron waves, periodically causing heavy ions to precipitate and produce Jupiter’s x-ray pulses. Our findings show that ion aurorae share common mechanisms across planetary systems, despite temporal, spatial, and energetic scales varying by orders of magnitude.

## Teaser

Jupiter’s unique x-ray aurora is driven by the same processes as Earth’s: Heavy ion scattering by electromagnetic waves.

## INTRODUCTION

Aurorae, observed from planetary polar regions across the solar system, are displays of light that are produced when energetic particles precipitate along magnetic field lines and transfer their energy to the atmosphere. Jupiter’s soft x-ray aurorae are produced by energetic [~megaelectron volts (MeV)] heavy ions (sulfur and oxygen), originally from the moon Io’s volcanic activities (*1*–*3*). The dynamic x-ray emissions often pulse with a regular beat of a few tens of minutes (*4*, *5*). The spectacular quasi-periodic auroral pulsations at Jupiter have also been observed in ultraviolet (UV), infrared, and radio emissions (*5*–*10*). The x-ray aurorae are predominately confined to the region poleward of Jupiter’s main aurora, connecting to Jupiter’s outer magnetosphere via magnetic field lines. The mapping of the emissions leads to the suggestion that the particle precipitations were driven by magnetic reconnection (*11*). However, observations show that the x-ray pulsations last for several Jupiter days or longer (*8*), evidencing that the driver may not be a transient process like magnetic reconnection.

To date, 40 years after their discovery, the mechanisms that cause these x-ray aurorae remain unknown. Simultaneous measurements of the magnetospheric environment and the auroral emissions are critical to revealing their driving mechanisms (*12*, *13*). Here, we present observations of Jupiter’s unique x-ray aurorae with simultaneous in situ measurements from the magnetosphere. In this study, we reveal the physical driver for Jupiter’s pulsating x-ray emissions by analyzing simultaneous in situ measurements from Juno and remote spectroscopic imaging by XMM-Newton telescope (XMM) during 16 and 17 July 2017. XMM’s European Photon Imaging Camera (EPIC-pn and MOS) instruments provided spatial, spectral, and timing data of Jupiter for a continuous 26-hour (~2.6 Jupiter rotations) observation from 18:26 UT on 16 July to 22:13 UT on 17 July, which was shifted to account for the ~46-min light travel time between Jupiter and Earth. This XMM observation was planned to coincide with the time when NASA’s Juno spacecraft was moving from 62 to 68 R_J_ (1 R_J_ = 71,492 km) radially away from the planet in the Southern Hemisphere in the predawn sector between ~0400 and 0430 magnetospheric local time (MLT).

Ionosphere-magnetosphere mapping from previous observations suggested that the origins of Jupiter’s x-ray auroral pulsations occurred at these distances from the planet (*4*). As described in Supplementary Text and figs. S1 and S2, Juno provided contemporaneous in situ measurements from the plasma sheet only when Jupiter’s north magnetic pole tilted to Earth. Therefore, we focus on the northern aurora, for which Juno’s in situ measurements detail what was happening in the plasma sheet during the x-ray pulses. At Jupiter, the analysis of these comparisons between in situ and remote sensing observations is more complex than at Earth. At Earth, during the time scale of an auroral event, typically tens of minutes, a spacecraft in the terrestrial magnetosphere (e.g., Cluster and THEMIS missions) usually travels little (e.g., hundreds of kilometers) in comparison to the spatial scale of a magnetospheric event (e.g., several Earth radii) that would cause a large auroral brightening so that this in situ spacecraft could be magnetically connected to the aurora region over the full auroral lifetime (*14*). This is not true for Jupiter, because the footprint of the aurora (which is rotating with Jupiter) with respect to Juno’s location changes substantially during an observation. There are also substantial travel times (a few tens of minutes) along the magnetic field expected from the outer magnetosphere to the Jovian aurora (*15*). Therefore, the correlation between a single outer magnetosphere event in Jupiter’s in situ measurements and a single auroral pulse cannot be expected on a one-to-one level basis. Instead, a series of successive events are required to draw reliable careful correlations, with the regular periodicity of the x-ray flares, providing an invaluable diagnostic signature of the source process.

## RESULTS

### A hypothesis of causality leading to the generation of x-ray aurora: Suggestive evidence from observations

**Fig. 1:**
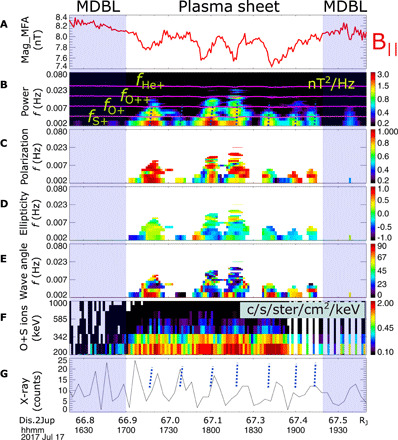
Juno and XMM measurement comparison. (**A**) Juno MAG measurements of the field-aligned magnetic component (B_∥_) in mean field–aligned coordinates (coordinates obtained over a 60-min window). (**B**) Power spectral density of the magnetic field perturbations with the gyro frequencies of various charge states of ions (He^+^, O^++^, O^+^, and S^+^) overlaid. (**C**) Degree of polarization of the waves. (**D**) Wave ellipticity. (**E**) Wave normal angle. (**F**) Juno JEDI measurements of the sulfur and oxygen energy and intensity. (**G**) XMM EPIC-pn and MOS light curves (binned to a resolution of 4 min) from the north x-ray aurora, which was observable at this time. The x-ray light curve has been shifted to account for the light travel time from Jupiter to XMM. Blue dashed lines show the times of EMIC waves. The time taken to precipitate along the magnetic field lines from the outer magnetosphere is expected to be tens of minutes or more so that it is not possible to directly connect a single EMIC wave with an x-ray pulse; however, they both exhibit similar quasi-periodicity.

Between 17:05 UT and 19:10 UT on 17 July, Juno encountered Jupiter’s outer magnetosphere plasma sheet but had not reached the central plasma sheet. Therefore, the magnetic field is dominated by the radial component. The plasma population is dominated by electrons and ions (protons, sulfur, and oxygen ions) that are injected by Io at ~6 R_J_ and gradually fill the magnetosphere outward from the Io plasma torus. Figure 1 shows the measurements of the magnetic field (*16*) and plasma (*17*) by Juno, together with an x-ray auroral light curve from XMM. The variation of the field-aligned magnetic component (B_∥_ in Fig. 1A) in the mean field–aligned coordinate system is indicative of a compressional mode wave (*18*). The two gray shadows highlighted correspond to the times when Juno was in the magnetodisc boundary layers, which are outside of the plasma sheet in the latitudinal direction. The magnetodisc boundary layers are characterized by strong magnetic field and depleted plasma content (see Supplementary Text and fig. S2). At the magnetic dips in Fig. 1A, the magnetic transverse wave power spectrum (Fig. 1B) shows enhancements around the frequencies of heavy ion gyro-periods, indicating that these waves are electromagnetic ion cyclotron (EMIC) waves associated with these ion species. The phase speed of EMIC waves is generally smaller than the Alfvén velocity (*19*). EMIC waves are often generated in the central plasma sheet. From there, they propagate along the magnetic field to the ionosphere so that the observations of EMIC waves are not restricted to this source region (*20*, *21*) but are more challenging to detect away from the plasma sheet. The degree of wave polarization (Fig. 1C) was generally greater than 70%, indicating that the waves are coherent signals (*22*). The wave normal angles (Fig. 1D) were generally small, and the wave ellipticity (Fig. 1E) mostly varied between the left-handed and linear modes, which are typical observational features of EMIC waves in geospace. Under ideal conditions, they are left-hand near–circularly polarized transverse waves that propagate at frequencies at or below the ion gyrofrequencies in the source region, but it is often observed that the waves have the left-handed to linear ellipticity (*23*, *24*), particularly in the outer magnetosphere where the magnetic field line is more nondipolar (*25*). Moreover, the direct detection of energetic heavy oxygen and sulfur ions in Fig. 1F shows coherent enhancements at the energies of several hundred kilo–electron volts (keV), matching the time instances of EMIC wave occurrences. These results suggest that these energetic heavy ions could be scattered by the intense EMIC waves, because these waves are known to be effective in scattering ions into field-aligned directions (*26*–*28*) and, through this scattering, to produce ion aurorae at Earth (*27*, *29*). We further test the interactions of EMIC waves with the heavy ions in Jupiter’s magnetosphere in the next section. The blue dashed lines in Fig. 1B highlight the discrete enhancements of EMIC wave power. The same dashed lines are marked in Fig. 1G to guide the comparison between EMIC wave pulsations and x-ray pulsations. The two pulsations are consistent, although not rigorously one-to-one connected. As explained above, this imperfect correlation is expected due to a combination of Jupiter’s rapidly rotating magnetosphere and wave travel time along the field.

In this study, Juno observed the EMIC waves in the boundary layer of the plasma sheet but not the central plasma sheet, from which the wave likely originated. EMIC waves form when there is an ion temperature anisotropy (when the perpendicular temperature is greater than the parallel temperature). The microinstability in generating EMIC waves has a maximum growth rate when the wave vector is parallel to the magnetic field (*30*), and the generated waves therefore propagate primarily parallel to the background magnetic field. Compressional waves have previously been found to modulate EMIC wave power in the Earth’s magnetosphere (*27*). A decreasing magnetic field in the negative cycle of a compressional wave would decrease Alfvén velocity and thus correspond to a lower minimum resonant energy. Therefore, more particles from lower energies may participate in the wave-particle interaction process, leading to the antiphase modulation (i.e., wave power peaks in magnetic dips) (*31*). In this study, EMIC waves were most intense during the magnetic troughs of the compressional wave shown in Fig. 1A and have the potential to scatter high-energy ions at measured energies of approximately hundreds of keV for precipitation along the magnetic field lines and toward the poles of the planet.

**Fig. 2:**
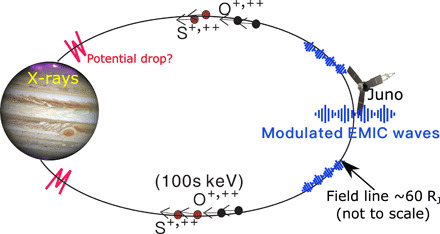
A schematic to illustrate the wave-particle interaction and the consequent x-ray emissions.

At Earth, the interactions between EMIC waves and ions generate Earth’s proton aurora (*32*). Jupiter’s x-ray aurora is known to be dominated by emissions from ions that collide with the Jovian neutral atmosphere and, through charge exchange, generate spectral lines that are characteristic of the precipitating heavy ions (*1*, *2*, *33*). A combination of oxygen and sulfur ions has been found to produce excellent fits to the observed x-ray auroral spectra (*2*, *33*, *34*). Figure S3 shows that this is the case for the aurora in this interval. We suggest that the ordering of Fig. 1 from A, B, and F to G therefore provides the chain of causality that leads to the generation of x-ray aurorae: Compressional mode waves trigger ion anisotropies that produce quasi-periodic EMIC waves, which further drive atmospheric precipitation of the energetic sulfur and oxygen ions along the magnetic fields via a pitch angle diffusion process to generate the x-ray auroral pulses observed. Figure 2 is a schematic, showing the interactions between the ultralow frequency (ULF) waves, EMIC waves, and heavy ions in the plasma sheet, which result in heavy ion precipitation into the ionosphere and produce soft x-ray emissions.

### Correlation between the compressional mode wave and x-ray flares over three planetary rotations

**Fig. 3:**
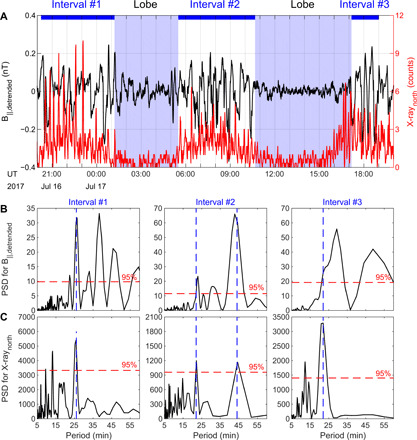
The comparison of periodicity for x-ray and magnetic field for 2.6 Jupiter rotations. (**A**) Detrended field-aligned magnetic field component (black) and northern x-ray emission (red). Lomb-Scargle periodograms of the detrended field-aligned magnetic field B_∥_ (**B**) and northern x-ray emission (**C**) for three intervals marked by the blue bars on the top. The red dashed lines indicate the confidence level of 95% for each analysis. The blue dashed vertical lines show shared x-ray and magnetic field power spectral density (PSD) periodogram peaks.

A global comparison spanning ~26 hours (2.6 Jupiter rotations) between compressional waves and x-ray flares is shown in Fig. 3. Both the magnetic field and northern x-ray emissions are presented at the time resolution of 1 min. As explained in Supplementary Text, the relative distance between Juno and the plasma sheet is modulated by the planetary rotation (*35*), as clearly evidenced by the particle data in fig. S2.

Figure 3 displays Lomb-Scargle periodograms, with periods expressed in minutes, of the detrended field-aligned magnetic field and x-ray counts for the three intervals marked on the top of panel (A). As guided by the vertical blue dashed lines, the magnetospheric compressional mode wave power and x-ray emissions pulse with a shared period for each of the viewing windows, supporting the physical connection described in Fig. 1. A periodicity of ~25 min is found in both datasets for the three intervals. During the second interval, an additional consistent periodicity is also found at ~45 min. These periodicities are fully compatible with previous studies on Jovian x-ray emissions (*5*, *8*, *36*, *37*) and also extensively reported in magnetospheric observations (*38*), e.g., radio waves (*39*), magnetic fields (*40*, *41*), and particles (*42*). Similar results are also obtained from wavelet analysis as shown in fig. S4. As highlighted by the red dashed ellipses in the two wave power spectra, x-ray emissions and compressional mode waves share common behaviors throughout the 26-hour x-ray observation, supporting the idea that the connection between the compressional mode waves, EMIC waves, energetic heavy ions, and x-ray emissions is systematic. It is noteworthy that the high-order harmonic periods of standing Alfvén waves could be 4 to 5 min (*40*, *43*), which is at the lower boundary of EMIC waves shown in Fig. 1. This reaches a crossover point between the frequencies associated with magnetohydrodynamic (MHD) waves and the gyrofrequencies of ions, where MHD can no longer apply. The 25- and 45-min compressional mode waves shown in fig. S4 may be associated with these standing Alfvén waves.

### Testing whether Jovian outer magnetospheric EMIC waves would generate auroral ion precipitation

**Fig. 4:**
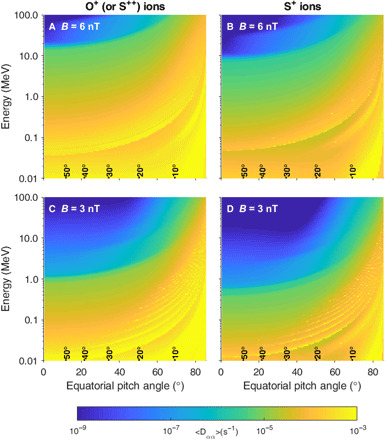
Local pitch angle diffusion coefficients Dαα for sulfur and oxygen ions. The diffusion coefficients Dαα are calculated using the PADIE code (see the Supplementary Materials) from a combination of H^+^, O^+^ (or S^++^), and S^+^ waves calculated locally at *B* ~ 6 and 3 nT, respectively, interacting with O^+^ (or S^++^) and S^+^ ions. The black numbers above the *x* axis show the maximum latitude a particle with this pitch angle will reach before mirroring. The left panels show the wave interaction with O^+^ (or S^++^) ions, and the right panels show the interaction with S^+^ ions.

The measurements from Juno and XMM show correlations between the magnetic compressional mode waves, EMIC wave power, energetic heavy ion fluxes, and x-ray pulsation rate, providing observational evidence of the magnetospheric driver for the ion precipitation that produces Jupiter’s x-ray aurora. We also note that at least two crucial processes are required from the magnetospheric source to x-ray aurora, which are (i) pitch angle scattering in the magnetosphere and (ii) particle acceleration to produce the MeV energies for the heavy ions. Figure 4 shows the local pitch angle diffusion coefficients calculated using the PADIE (Pitch Angle and energy Diffusion of Ions and Electrons) code (*44*) using the input from the EMIC intensity obtained by Juno (Fig. 1B). PADIE calculates the particle diffusion coefficients due to their resonant interactions with various plasma waves by simultaneously solving the wave-particle resonance condition and the wave dispersion in a cold plasma with a magnetic field. The modeling results show that EMIC waves observed by Juno would efficiently (the yellow color) scatter oxygen and sulfur ions with energies over a large energy range (i.e., from 10 keV to 1 MeV). Results for *B* = 6 nT are shown in the top panels, and results for *B* = 3 nT are shown in the bottom panels (where *B* is the background magnetic field intensity), indicating that the pitch angle scattering process is efficient under varied conditions of background magnetic strengths. The interaction is much weaker near the central plasmadisc, e.g., *B* = 1 nT (please see fig. S5). As shown in Fig. 1F, the energy of the enhanced ion flux was up to 500 keV, which has a strong interaction between EMIC waves and ions with equatorial pitch angles <70°. Therefore, the modeling results confirm the capability of the pitch angle scattering process with the observed waves. Moreover, it is known that EMIC waves can efficiently scatter relativistic electrons with energies above MeV at Earth (*45*, *46*), which may contribute to simultaneous x-ray and UV aurora flares (*8*) at Jupiter.

It is important to note that the Juno spacecraft was relatively far from the central plasma sheet during these observations, as evidenced by the large magnetic B_∥_ component (~8 nT) compared to the central plasma sheet (~1 to 4 nT). By conserving the first adiabatic invariant, the particles measured by Juno would have pitch angles <45° at the magnetic equator. The observed heavy ions with relatively small equatorial pitch angles were probably the consequence of wave-particle interactions, which scattered them to smaller pitch angles and led them to travel to higher latitudes away from the central plasma sheet. The pitch angle diffusion coefficients in Fig. 4 and fig. S5 confirm that the ions can undergo a diffusive process of scattering from very high pitch angles all the way to field-aligned pitch angles (i.e., into the loss cone).

## DISCUSSION

In this study, a clear correlation is identified between compressional mode waves, EMIC waves, and x-ray auroral emissions. It remains unclear what systematically drives the compressional mode waves in Jupiter’s outer magnetosphere. Solar wind compressions are known to produce compressional mode waves in the magnetosphere (*47*). Magnetopause surface waves could be a potential driver for producing systematic compressional waves at a large spatial scale on the dayside magnetopause (*48*, *49*). Alternatively, high-speed plasma flows from the Vasyliunas cycle (*50*) could also potentially produce compressional waves, as has been found to drive the transient aurora at Saturn (*51*). Beside the macrophysics processes in producing compressional waves, the microphysics mechanism is also compelling. The temperature anisotropy (i.e., perpendicular temperature is greater than the parallel temperature) not only provides an energy source for producing EMIC waves but also could be a source to generate compressional mode waves, for example, mirror-mode or slow-mode waves (*52*).

Our study raises important questions about the mapping and origins of Jupiter’s aurora. The x-ray-EMIC wave correlations observed in this study occur in the predawn sector of Jupiter’s outer magnetosphere. Previous studies have tried to map the source location for the x-rays, and while the radial distances in this study are well matched to those expected, the local time location is not consistent with previous mapping (*4*). It may well be that Jupiter’s polar magnetic field is more twisted than expected and that the local times are therefore shifted beyond the expectations from the current mapping. Recent numerical simulation results show that Jupiter’s near-pole magnetic fields are closed and helical (*53*), which may trigger pulsating precipitation via wave-particle interactions.

The EMIC waves shown here may be excited in many local time sectors in the outer magnetosphere. If they are found in all local time sectors, then the localized nature of Jupiter’s x-ray auroral flares (*5*) is likely determined by other localized features. Previous studies have proposed that large potential drops would be required to accelerate particles to produce the x-ray aurora (*54*). It is possible that the periodic precipitations of heavy ions are accompanied by periodic or quasi-steady accelerations. Near-pole potential drops in specific locations offer a plausible mechanism in producing the MeV accelerations of heavy ions required for the observed x-ray auroral emissions. These megavolt potential drops have been observed in Jupiter’s polar region (*55*, *56*). The magnetic field in Jupiter’s north pole is known to be highly nondipolar (*57*, *58*), and this would produce a variety of different mirror forces and associated potential drops and locations suitable for particles to drift into a loss cone. This can also potentially explain observed north-south asymmetries (*4*), because the magnetic field and potential drops are found to be significantly different in the northern and southern polar regions (*59*). The important role of near-pole potential drops may well also be the reason that Jupiter has x-ray aurorae, while Saturn has no such emissions. Therefore, while the compressional mode waves and EMICs may drive the quasi-periodically pulsed precipitation of ions into the polar region, it is likely that additional acceleration may be required for these ions to become sufficiently energetic to generate the observed x-ray aurora.

At Earth, EMIC waves are mostly excited in the inner magnetosphere near the plasmasphere and can produce the proton aurora at relatively low latitudes, for example, in the subauroral region (*29*, *32*, *60*). This study reveals that EMIC waves could drive important ion dynamics not only in Jupiter’s inner magnetosphere (*61*) but also throughout Jupiter’s middle and distant magnetosphere, producing the spectacular x-ray pulsations in Jupiter’s high-latitude polar regions. Our results demonstrate that the composition of plasma sources is crucial in driving planetary emissions. The understanding of the connection between planetary magnetospheric processes and x-ray emissions also sheds light on the processes in many thermally dominated plasmas across the universe, because the fundamental interaction processes between EMIC waves and ions are analogous in different planets regardless of the large variety of their magnetized environments (*32*, *62*–*64*).

## MATERIALS AND METHODS

### Fitting the spectra with atomic charge exchange models

Figure S3 shows Jupiter’s northern auroral x-ray spectrum from 16 and 17 July 2017, as observed with XMM’s EPIC-pn instrument. The spectrum includes the characteristic bump from 0.5 to 0.7 keV, which indicates oxygen charge exchange emission (*8*, *65*, *66*). To produce these high charge states, the oxygen ions must have energies in excess of about 1 MeV per atomic mass unit. Below 0.4 keV, there are a forest of spectral lines. Charge exchange lines from sulfur provide an excellent fit to this region, and theoretical arguments also support sulfur ion production of the emission in this region (*67*, *68*). However, we note that the spectral resolution at these energies is low, and it is possible to also attain fits to the spectrum below 0.4 keV by replacing sulfur ions in the model with a combination of other ion species (e.g., carbon).

### Modeling the local pitch angle diffusion coefficient Dαα

The local pitch angle diffusion coefficient Dαα is calculated using the PADIE code (*45*). PADIE uses quasi-linear theory to treat wave-particle interactions as a diffusion process (*44*). To calculate the diffusion coefficients, PADIE solves the wave-particle resonance condition in conjunction with the cold plasma wave dispersion. In this case, we use local diffusion coefficients calculated at one particular latitude to demonstrate that the EMIC waves observed here can resonate with a wide range of relevant ion energies and have a substantial pitch angle diffusion effect to scatter ions into the loss cone.

The following input values were used: test magnetic latitude is 2° and background magnetic field strength is assumed to be 1, 3, or 6 nT. The plasma density is set to be 0.02 cm^−3^ based on previous statistical survey (*69*), and the ion composition is set to be 22% H^+^, 56% O^+^, and 22% S^+^, following previous literature (*70*, *71*). The average magnetic wave amplitude is taken to be 1 nT, with the wave frequency centered on 0.004 Hz (width of 0.003 Hz and cutoffs at 0.0001 and 0.027 Hz), following the observations from Fig. 1B. The wave normal angle is assumed to be field-aligned (peak at 0°, width of 15°, and upper and lower cutoffs at 0° and 45°). The diffusion coefficient is summed over harmonic resonance number, *n*, from −10 up to and including +10.

## SUPPLEMENTARY MATERIALS

Supplementary material for this article is available at http://advances.sciencemag.org/cgi/content/full/7/28/eabf0851/DC1
